# A Comparison of Clinical Outcomes of Dislocated Intraocular Lens Fixation between In Situ Refixation and Conventional Exchange Technique Combined with Vitrectomy

**DOI:** 10.1155/2016/5942687

**Published:** 2016-03-29

**Authors:** Sun Jung Eum, Myung Jun Kim, Hong Kyun Kim

**Affiliations:** Department of Ophthalmology, Kyungpook National University School of Medicine, 50 Samduk-2-ga, Jung-gu, Daegu 700-721, Republic of Korea

## Abstract

*Purpose*. To evaluate surgical efficacy of in situ refixation technique for dislocated posterior chamber intraocular lens (PCIOL).* Methods*. This was a single-center retrospective case series. 34 patients (34 eyes) who underwent sclera fixation for dislocated IOLs combined with vitrectomy were studied. Of 34 eyes, 17 eyes underwent IOL exchange and the other 17 eyes underwent in situ refixation.* Results*. Mean follow-up period was 6 months. Mean logMAR best corrected visual acuity (BCVA) was not significantly different between the groups 6 months after surgery (0.10 ± 0.03 in the IOL exchange group and 0.10 ± 0.05 in the refixation group; *p* = 0.065). Surgically induced astigmatism (SIA) was significantly lower in the refixation group (0.79 ± 0.41) than in the IOL exchange group (1.29 ± 0.46) (*p* = 0.004) at 3 months, which persisted to 6 months (1.13 ± 0.18 in the IOL exchange group and 0.74 ± 0.11 in the refixation group; *p* = 0.006). Postoperative complications occurred in 3 eyes in the IOL exchange group (17.6%) and 2 eyes in the refixation group (11.8%). However, all of the patients were well managed without additional surgery.* Conclusion*. The in situ refixation technique should be preferentially considered if surgery is indicated since it seemed to produce a sustained less SIA compared to IOL exchange.

## 1. Introduction

Dislocation of the intraocular lens (IOL) after cataract surgery has been reported to occur in 0.2% to 1.8% of the patients [1, 2]. This uncommon ocular complication is important because it leads to serious visual disturbance that may need complicated surgical correction. IOL dislocation during the early postoperative period occurs because of inadequate capsular bag or ciliary sulcus support, whereas optic or haptic induced capsular damage can lead to IOL dislocation at a later stage [3, 4]. A variety of techniques for managing dislocated IOLs have been reported which can generally be classified into open- and closed-eye procedures [5–12]. Extraction of the dislocated IOL in the open eye method involves removal of a dislocated IOL through a large corneal incision followed by exchanging with a new secondary IOL. It accompanies the risk of vitreous prolapse, ocular collapse, intraocular hemorrhage, and induction of large amounts of astigmatism. Repositioning of the dislocated IOL using a closed-eye method is a desirable alternative [11]; however, it entails disadvantages such as surgical difficulty and multiple instrument passages. During surgical intervention, an important consideration is whether to remove, exchange, or reposition the dislocated IOL. The decision to undertake exchange or refixation of a dislocated IOL is usually made based on the clinical features of an individual case. If dislocated IOL is not adequate for reposition, it may be removed and exchanged. However, when there is no contraindication to reposition and sclera fixation of dislocated original IOL, the patient may receive a sclerally sutured IOL. Several previous studies have already identified improved best corrected visual acuity (BCVA) after sclerally fixated sutured posterior chamber intraocular lens (PCIOL) in patients with dislocated IOLs [1, 4, 12]. However, postoperative outcomes may differ according to the surgical techniques.

The aim of this study was first to introduce in situ refixation technique, which is a novel repositioning technique using a bilimbal small incision to manage posteriorly dislocated IOL, and then to evaluate its surgical efficacy in a retrospective comparative study.

## 2. Materials and Methods

### 2.1. Subjects

The study protocol was approved by the Institutional Review Board of the Kyungpook National University School of Medicine. A retrospective review was conducted on the medical records of 34 eyes of 34 patients with dislocated IOLs who underwent IOL exchange or in situ refixation combined with vitrectomy between January 2010 and May 2015. All surgeries were performed by one surgeon (H. K. Kim) at Kyungpook National University Hospital, Daegu, Republic of Korea. Patients aging 18 or older, who have suffered dislocated IOL without ample capsular support and so have to undergo scleral fixation surgery of dislocated IOL, were included. The exclusion criteria were as follows: (1) history of underlying corneal disease (e.g., corneal laceration, bullous keratopathy, or Fuchs' dystrophy); (2) glaucoma; (3) history of optic neuritis; (4) state of aphakia; (5) history of previous IOL dislocation; and (6) follow-up duration less than 6 months.

The 34 patients in the study were divided into two groups based on the surgical techniques for managing dislocated IOLs: the IOL exchange group and the in situ refixation group. The patients in the IOL exchange group underwent removal of dislocated IOL and concurrent secondary IOL implantation with scleral fixation. The patients in the in situ refixation group underwent repositioning of the dislocated IOL using a bilimbal small incision with double haptic scleral fixation. The surgical technique was chosen with the following considerations: IOL design, kinds of optic material, presence of deformation of the IOL, and necessity of refractive change. Patients with dislocated IOL, which was 3-piece design without any deformation, were chosen for in situ refixation technique. Patients, who had late haptic or single piece designed IOL, any deformation or breakdown of IOL, and the needs for refractive correction, were undergone with IOL exchange technique.

### 2.2. Surgical Technique

All patients underwent vitrectomy for anteriorly prolapsed vitreous before the scleral fixation of IOL under general anesthesia. Pars plana approach or anterior two-port vitrectomy were performed. Vitrectomy techniques were chosen at the surgeon's discretion according to the states of dislocated IOLs and vitreoretinal pathology. Pars plana approach was performed in cases with complete IOL dislocation into the vitreous cavity or dislocation posteriorly with one haptics adherent to the vitreous base. A 23-gauge standard three-port vitrectomy was setup to remove the vitreous and free the dislocated IOL from vitreous adhesions. Following placement of the infusion cannula, two sclerotomies were placed through the pars plana. In each cases of PPV, the surgeon attempted a complete vitrectomy that extended to the periphery to remove as much vitreous as possible, because the residual vitreous might induce IOL kinking and vitreoretinal traction afterward. After core vitrectomy, the peripheral retina was carefully examined with sclera indentation to remove the vitreous gel and find any retinal break. Two 23-gauge peeling forceps were introduced into the vitreous cavity through the two previously positioned port sites to grasp and raise the dislocated IOL up to the back of the iris plane so the IOL could be clearly visualized. After PPV, sclerotomy sites were carefully checked for any vitreous incarceration. In contrast, when prolapsed vitreous was present in the anterior chamber, AV was performed with introducing a bimanual port through a corneal incision site to remove and prevent traction on vitreous strands.

**Figure 1:**
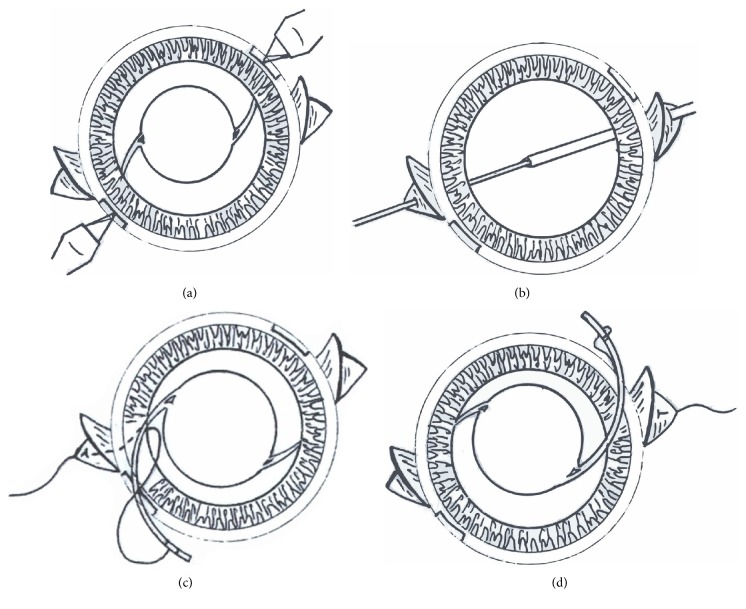
Drawing images of in situ refixation technique. (a) Bilimbal incision was made on the nearest axis of the dislocated haptics. (b) The suture needle was inserted under the two triangular partial thickness sclera flaps. (c) One haptics was externalized through the limbal incision and one of the cut suture thread ends was tied to the haptics. (d) The other haptics was tied with the same procedure to that in (c).

After vitrectomy, an IOL was sclerally fixated with the two different surgical methods: conventional IOL exchange or IOL refixation. A conjunctival incision was created and two-half thickness triangular scleral flaps with 180° apart were performed. In the IOL exchange group, a slit knife was used to make an approximately 6.0 mm superior corneal incision. In contrast, 1.5 mm sized two limbal incisions on opposite sides of direction were made in the IOL refixation group (Figure 1(a)). We named this novel approach as the in situ refixation technique. The dislocated IOL is visualized at the back of the iris plane after being floated from the vitreous cavity through vitrectomy procedure. The anterior chamber was maintained using an ophthalmic viscosurgical device (OVD) (sodium hyaluronate 1.65%, chondroitin sulfate 4% [DiscoVisc]) during scleral fixation. A 10-0 polypropylene (PROLENE) suture was inserted with a curved needle under the scleral flap about 1.5–2.0 mm posterior to the limbus and it was pulled out to the opposite sclera flap (Figure 1(b)). In the IOL refixation group, the suture thread was hooked out of the eye through limbal incision site and cut in two pieces. Each haptics was externalized through one of the limbal incision sites, and the cut suture threads were tied to each haptics (Figures 1(c) and 1(d)). Once the suture was tied and tensed to the haptics, it was reinserted intraocularly. After tightening sutured haptics, centration of the lens was carefully checked and the sutures were tied under the scleral flaps. A stromal hydration was performed at both edges of the two limbal incision sites instead of suture to help seal it. In the IOL exchange group, a conventional IOL exchange technique was applied to scleral fixation of the IOL. When we used IOL cutter or refolding technique for the removal of dislocated IOL, we made 3.5 mm superior limbal incision. In cases of rigid optic material, such as poly(methyl methacrylate) (PMMA), 6 mm sized superior scleral tunnel incision was used. The dislocated original IOL was grasped with an intraocular forceps and carefully extracted through superior incision. After the dislocated IOL was removed, a retrieved suture was pulled out through the same site with H-hook and cut. In all patients in the IOL exchange group, a new secondary IOL was chosen as Model MN60AC (Alcon Laboratories, Inc.), which is a foldable 3-piece acrylic IOL. Knots were buried under the scleral flaps. The corneal incision site was sutured using 10-0 ETHILON and conjunctival suture was made with 8-0 vicryl.

### 2.3. Main Outcome Measures

All patients underwent a comprehensive ophthalmological examination on their scheduled follow-up date. The following parameters were included before and after surgery: age, gender, BCVA, intraocular pressure (IOP), endothelial cell density, and spherical equivalent as determined by biometry using an autorefractometer (Topcon, KR-8800, autokeratorefractometer, Tokyo, Japan) and IOL master (Carl Zeiss Meditec, Jena, Germany). Surgically induced astigmatism (SIA) and surgical complications were also assessed. Potential postoperative complications included marked IOP elevation higher than 25 mmHg, corneal decompensation, IOL redislocation or capture, suture knot exposure, cystoids macular edema (CME), vitreous hemorrhage, retinal break or detachment, and endophthalmitis. Visual acuities were measured with Snellen's chart, and values were converted to the logarithm of the minimum angle of resolution (logMAR). SIA was calculated by the astigmatic vector analysis [13, 14]. Corneal endothelial cell density (cells/mm^2^) was inspected in central corneal endothelial cells with a noncontact specular microscope (Topcon Corp., SP-3000P, Japan), and analyzed by manual check of the automatic analysis software before the operation and 3 months after the operation. Macular optical coherence tomography (OCT) (Carl Zeiss Meditec, Dublin, CA) was performed in the case of the presence of metamorphopsia or reduced BCVA during the follow-up. All patients in this study underwent PPV or AV before the sclera fixation of IOL. Undergoing PPV may influence the surgical outcomes and induce the difference among patients so the subjects were also classified into two groups according to the surgical option of vitreous management. The PPV group included patients with sclera fixation of IOL who underwent full vitrectomy, and the AV group comprised patients with sclera fixation of IOL who underwent AV only.

Statistical analysis was performed using SPSS software version 18.0 (SPSS, Inc., Chicago, IL, USA). The relationship between the IOL exchange and IOL refixation groups was compared using Student's *t*-test. Preoperative and postoperative parameters were compared using paired *t*-test. The distributions for variables were expressed as mean ± standard deviation. Statistical significance was defined as *p* value < 0.05 for all tests.

## 3. Results

### 3.1. Patients Demographics

In total, 34 eyes of 34 patients were included in this study. The subjects included 29 men and 5 women, ranging in the age from 40 to 79 years. The mean postoperative follow-up was 8.3 months (range: 6–12 months). Of 34 eyes, 17 eyes (50%) were assigned to the IOL exchange group and 17 eyes (50%) were assigned to the IOL refixation group.  Table 1 summarized the demographic characteristics of the patients. There was no significant difference in terms of preoperative age, axial length, and postoperative follow-up duration between the two groups. The cause of IOL dislocation seemed to be eye trauma in four eyes, pseudoexfoliation syndrome in three eyes, inadequate capsular support after neodymium:yttrium-aluminum-garnet (Nd:YAG) capsulotomy in three eyes, inadequate capsular or zonular support in the absence of Nd:YAG capsulotomy in four eyes, and unknown cause in 20 eyes. These data are shown in Table 2. The preoperative underlying ocular diseases in the IOL exchange group were previous rhegmatogenous retinal detachment in 3 eyes (2 eyes underwent PPV and 1 eye underwent segmental scleral buckle procedure) and diabetic retinopathy in 2 eyes. The IOL refixation group included 1 vitrectomized eye owing to previous retinal detachment, 1 eye with diabetic retinopathy, and 1 eye with previous branch retinal vein occlusion history.

### 3.2. Visual Outcomes

Visual outcomes and SIA are presented in Table 3. Mean BCVA (logMAR) significantly improved from 0.35 ± 0.24 preoperatively to 0.11 ± 0.08 postoperatively at 3 months in the IOL exchange group and from 0.31 ± 0.20 preoperatively to 0.10 ± 0.08 postoperatively in the IOL refixation group (*p* < 0.001, for both groups). However, both preoperative and postoperative visual results were similar between the eyes that underwent IOL exchange and the eyes that underwent IOL refixation (*p* = 0.613 and *p* = 0.790, resp.). No statistically significant difference was found between BCVA at 3 months and BCVA at 6 months (0.10 ± 0.03) (*p* = 0.096) in IOL exchange group and IOL refixation group (0.10 ± 0.05) (*p* = 0.065). Notably, the IOL refixation group exhibited significantly less SIA (0.79 ± 0.41) compared to the IOL exchange group (1.29 ± 0.46) 3 months after surgery (*p* = 0.004). This significant difference in SIA persisted to 6 months (1.13 ± 0.18 in the IOL exchange group and 0.74 ± 0.11 in the refixation group; *p* = 0.006).

In the IOL exchange group, the mean spherical equivalent (diopter) changed from 2.34 ± 6.84 to −0.73 ± 1.29 (*p* = 0.083), while, in the IOL refixation group, the parameter significantly improved from 2.80 ± 5.97 to −1.18 ± 0.96 (*p* = 0.02). The preoperative and postoperative mean spherical equivalents were similar between the two groups (*p* = 0.842 and *p* = 0.271, resp.).

We also analyzed visual outcomes of the two groups classified according to the surgical method of vitreous management: PPV or AV. Mean preoperative BCVA (logMAR) were 0.42 ± 0.63 in PPV group and 0.33 ± 0.30 in AV group (*p* = 0.56). Mean postoperative BCVA (logMAR) were also similar between the PPV group (0.21 ± 0.21) and the AV group (0.10 ± 0.14) (*p* = 0.08).

### 3.3. Safety Outcomes

Both groups showed a significant decrease in postoperative endothelial cell density compared to the density before surgery (*p* = 0.003 in the IOL exchange group and *p* = 0.015 in the IOL refixation group, resp.). However, no significant between-group difference was found before surgery (*p* = 0.232) and at 6 months after surgery (*p* = 0.612) (Table 4). IOP elevation over 25 mmHg occurred in 2 out of 34 eyes (5.9%) from the first day after surgery. Elevated IOP was well controlled with antiglaucoma topical medication, and IOP was maintained within the normal range at the final visit time. Furthermore, the two groups showed reduction of IOP from 17.1 ± 4.7 preoperatively to 16.5 ± 2.8 postoperatively in the IOL exchange group and from 16.0 ± 3.3 preoperatively to 14.8 ± 3.0 postoperatively in the IOL refixation group. The between-group difference was not statistically significant before surgery (*p* = 0.747) and after surgery (*p* = 0.230). In addition, the IOP reduction was not significantly different between the two groups (*p* = 0.421 and *p* = 0.163, resp.) (Table 5). Intraoperative complications were not observed in either group. Postoperative complications developed in 3 eyes (retinal break, transient vitreous hemorrhage, IOP elevation) that underwent IOL exchange and 2 eyes (pupillary optic capture of IOL, IOP elevation) that underwent IOL refixation. One retinal break case was treated with laser photocoagulation and had no further complication. One case of vitreous hemorrhage developed in the IOL exchange group, but it was transient and resolved at the final visit without needing additional vitreous surgery. Pupillary optic capture of IOL developed in one eye that underwent IOL refixation. After pupil dilatation, the patient remained in a supine position and optic capture resolved spontaneously. The patient did not need any further procedure. Other postoperative complications, such as redislocation of IOL, CME, retinal detachment, hypotony, secondary glaucoma, and infective endophthalmitis, were not observed.

## 4. Discussion

In our retrospective study, the results showed that the in situ refixation technique had less SIA than IOL exchange at 3 months and it persisted at 6-month postoperative follow-up time. The IOL exchange method requires a larger corneal incision to remove dislocated IOL, but the in situ refixation technique minimized the cornea incision size.

Therapeutic options were typically decided based on the clinical features of individual cases. A variety of methods for managing dislocated IOL have been reported, including observation, IOL exchange, and IOL refixation [5–11]. Several comparative clinical studies have been reported, but relatively few studies have considered postoperative outcomes, particularly in terms of SIA after IOL scleral fixation surgery. Theoretically, IOL refixation is the optimal surgical option because it is less traumatic than extracting the dislocated IOL and it provides structural stability [8]. In the IOL exchange technique, extraction of the dislocated IOL with an open-system method carries the risk of ocular structural damage, vitreous prolapse, hypotony, and corneal astigmatism induced by a large corneal wound [11]. Therefore, IOL refixation using a closed-eye method is a more preferred surgical technique if it can be performed with intact haptics [6]. Oh et al. [15] reported that there was no significant difference in SIA between IOL exchange and IOL refixation groups despite the considerable SIA by corneal incision performed during surgery in the IOL exchange group. However, our study showed significantly less SIA in the IOL refixation group at postoperative 6 months. Bilimbal incision for haptic externalization might have an impact on lowering SIA when compared to the sutureless IOL fixation method of the previous study [15].

Notably, the most common postoperative complication was a significant decrease in endothelial cell density in both the IOL exchange and the IOL refixation groups. Wang et al. [16] reported that corneal endothelial cell density decreased remarkably after IOL exchange or refixation surgery without a significant difference in the decrease between the two groups. We initially predicted that the IOL refixation group would have less decrease in endothelial cell density than the IOL exchange group because the smaller incision would cause less trauma to the corneal endothelium. However, all patients in the two groups underwent scleral fixation and the loss of endothelial cell density might be attributable to this increased surgical manipulation. Similar to the previous studies, our results showed significantly decreased endothelial cell density in both groups, but there was no statistically significant difference between the two groups.

There was 1 case of IOP elevation in each group during the follow-up period. Increased IOP occurred in 5.9% of the patients who underwent IOL exchange and IOL refixation, respectively, which is a relatively lower incidence compared to the previous studies [9, 17]. Although we excluded patients with preexisting glaucoma in this study, the reason for a lower incidence of IOP elevation remains to be explained. IOP increase in two patients was well managed with IOP-lowering medication within 1 month after surgery, so IOP elevation may not affect the final functional outcome in the long term. During the follow-up time, both groups showed slight decrease of mean IOP at final visit compared to preoperative values. There was no patient who presented hypotony or sclerotomy site leakage. Although the magnitude of IOP reduction was not statistically significant either IOL exchange or IOL refixation group, the reason for IOP lowering effect after surgery should be further studied for a longer period. The IOL refixation group had 1 case of pupillary optic capture of IOL, and it was spontaneously resolved with pupil dilatation and position change. Moreover, 1 case of postoperative retinal break and transient vitreous hemorrhage occurred in the IOL exchange group. Sclerally fixated IOL implantation in the posterior capsule carries the risk of vitreous hemorrhage and retinal breaks with consequent retinal detachment [5]. In spite of these postoperative vitreoretinal complications, several studies have reported acceptable safety for the procedure [18]. In our study, retinal break was readily treated with laser photocoagulation. Vitreous hemorrhage was resolved with conservative treatment and did not require additional retinal surgery. Bellamy has reported that 22% of eyes that underwent PPV with IOL removal and exchange to open loop anterior chamber IOL presented CME [19]. In this study, no patient presented CME during follow-up period. Minimal incision and careful manipulating IOL while extracting it through incision site, especially avoiding contact with uvea, might induce this result. Suture-related complications, such as knot exposure, suture degradation or breakage, and IOL decentration or tilting were not found in either the IOL exchange or the refixation group. A significantly improved final mean BCVA was achieved in both groups, but the between-group difference was not statistically significant.

Surgical techniques should be selected based on the ophthalmological features of individual patients with regard to the status of the dislocated IOL, adequate capsular support, and concurrent ocular complications. In this study, the most important factor for consideration was the status of the dislocated IOL, such as damaged or highly flexible haptics that were unsuitable for adequate suture support and the size and material of optics. The postoperative results showed that the IOL refixation group had a lower magnitude of SIA than the IOL exchange group. Although IOL exchange technique has the advantage of being useful no matter the type of IOL and degree of dislocation, it necessarily leads to a large corneal incision and SIA [20]. Removal of the dislocated IOL through a large corneal incision site is also accompanied by the possibility of vitreous prolapse, cornea endothelium and iris damage, hypotony, and retinal damage, such as retinal break or retinal detachment [21, 22]. In contrast, IOL refixation has the advantage of leading to relatively less SIA than IOL exchange, owing to the small incision size maintaining structural stability. However, this surgical technique has limited indication because it can only be conducted in case of intact haptics with adequate suture support. This technique has disadvantage for the difficulty in manipulating the haptics while extracting it through bilimbal incision site. IOL refixation includes scleral sutured PCIOL procedure so it also has comparable potential complication like other sclera refixation, such as IOL redislocation or capture, suture breakage or suture knot exposure, vitreous hemorrhage, and CME. Smiddy et al. [8, 23] previously reported that refixation of dislocated IOL into the ciliary sulcus using residual capsule for support is the most commonly used surgical technique. This nonsuturing technique is the least traumatic to the ocular structure compared to fixating the IOL into the sclera by suture because it avoids excessive surgical manipulation. However, it can be performed for selected patients who have adequate residual peripheral capsular support. In patients with a lack of suitable capsular support, scleral suture fixation of an IOL is a good alternative surgical option [24]. Numerous methods are currently used for transscleral fixation of IOLs and each technique has its advantages and drawbacks. Hoffman et al. [25] reported modified sclera fixation technique using a sclera pocket through a clear corneal incision which avoids the need for conjunctival dissection or sclera cautery. Scharioth et al. [26] reported sutureless intrascleral PCIOL fixation technique using a limbus-parallel tunnel of 50% sclera thickness starting from the ciliary sulcus sclerotomies without the need for suturing procedures. Previously reported studies have disadvantages for potential complications of suture erosion, suture-knot exposure, and recurrent dislocation. Despite a variety of surgical techniques for managing dislocated IOLs, a definitive surgical technique for dislocated IOL rescue has not yet been suggested. The surgical option is generally decided based on a surgeon's best judgment given an individual patient's characteristics, and it usually provides significantly improved visual acuity without serious irreparable postoperative complications.

In this study, we also reorganized the patients into two groups according to the surgical method of vitreous management as PPV group and AV group. A previous study [10] reported similar degree of visual improvement in patients who underwent sclera fixation of PCIOL with PPV or AV. This study represents comparable visual outcomes between two groups which is consistent with previous study.

Limitations of this study include analyses from retrospective design and small number of cases (34 eyes) with relatively short follow-up periods (6 months). This study includes lack of measurements with evaluating astigmatism using corneal topography or Scheimpflug imaging. Furthermore, we did not consider the effect of PPV except for preoperative and postoperative BCVA. Previous studies have reported that the performance of a combined PPV has an impact on a more complicated condition [27]. Future studies with larger scales in patients and longer follow-up with evaluating SIA from various methods are highly recommended to confirm true statistical significant difference.

In conclusion, in situ IOL refixation is a beneficial surgical technique in IOL dislocation, producing less SIA compared to IOL exchange with scleral fixation. The two groups had similar results for BCVA, IOP, endothelial cell density, and postoperative complications, with no significant difference at the final follow-up visit. Therefore, IOL refixation technique can be the preferred surgical option because it provides early visual rehabilitation in patient with a dislocated IOL but no damage in haptics. In situ IOL refixation for managing IOL dislocation can produce significantly increased BCVA with less SIA than IOL exchange.

## Figures and Tables

**Table 1:** Demographic characteristics of patients with IOL exchange and IOL refixation groups.

	IOL exchange	IOL refixation
Number of patients (eyes)	17 (17)	17 (17)
Age (years)	56.92 ± 11.36	63.65 ± 9.93
Gender (male/female)	12/5	17/0
Right/left	7/10	9/8
Axial length (mm)	24.20 ± 1.43	24.29 ± 1.84

**Table 2:** Presumed causes of IOL dislocation.

	IOL exchange	IOL refixation
Eye trauma	2 (12%)	2 (12%)
Pseudoexfoliation syndrome	2 (12%)	1 (6%)
Nd:YAG capsulotomy	2 (12%)	1 (6%)
Inadequate capsular/zonular support	2 (12%)	2 (12%)
Unknown	9 (52%)	11 (64%)

**Table 3:** Visual outcomes and SIA after scleral fixation of IOL.

	IOL exchange	IOL refixation	*p*
Preoperative BCVA, logMAR	0.35 ± 0.24	0.31 ± 0.20	0.613
Postoperative 3-month BCVA, logMAR	0.11 ± 0.08	0.10 ± 0.08	0.790
SIA at 3 months	1.29 ± 0.46	0.79 ± 0.41	0.004
SIA at 6 months	1.13 ± 0.18	0.74 ± 0.11	0.006

SIA: surgically induced astigmatism; IOL: intraocular lens; BCVA: best corrected visual acuity.

**Table 4:** Comparison of endothelial cell density between the IOL exchange and IOL refixation group before and after surgery.

	IOL exchange	IOL refixation	*p*
Before operation	2070.4 ± 458.8	1778.5 ± 775.6	0.232
Postoperative 6 months	1805.9 ± 426.5	1689.6 ± 685.7	0.612
*p*	0.003	0.015	

IOL: intraocular lens.

**Table 5:** Comparison of IOP between the IOL exchange and IOL refixation group before and after surgery.

	IOL exchange	IOL refixation	*p*
Before operation	17.1 ± 4.7	16.0 ± 3.3	0.747
Postoperative 6 months	16.5 ± 2.8	14.8 ± 3.0	0.230
*p*	0.421	0.163	

IOP: intraocular pressure; IOL: intraocular lens.
